# Pf16 and phiPMW: Expanding the realm of *Pseudomonas putida* bacteriophages

**DOI:** 10.1371/journal.pone.0184307

**Published:** 2017-09-06

**Authors:** Damian J. Magill, Victor N. Krylov, Olga V. Shaburova, John W. McGrath, Christopher C. R. Allen, John P. Quinn, Leonid A. Kulakov

**Affiliations:** 1 Queen's University Belfast, School of Biological Sciences, Medical Biology Centre, Belfast, Northern Ireland; 2 Department of Microbiology, Laboratory for Genetics of Bacteriophages, I.I. Mechnikov Research Institute for Vaccines and Sera, Moscow, Russia; Centro Nacional de Biotecnologia, SPAIN

## Abstract

We present the analysis of two novel *Pseudomonas putida* phages, pf16 and phiPMW. Pf16 represents a peripherally related T4-like phage, and is the first of its kind infecting a Pseudomonad, with evidence suggesting cyanophage origins. Extensive divergence has resulted in pf16 occupying a newly defined clade designated as the pf16-related phages, lying at the interface of the Schizo T-Evens and Exo T-Evens. Recombination with an ancestor of the *P*. *putida* phage AF is likely responsible for the tropism of this phage. phiPMW represents a completely novel *Pseudomonas* phage with a genome containing substantial genetic novelty through its many hypothetical proteins. Evidence suggests that this phage has been extensively shaped through gene transfer events and vertical evolution. Phylogenetics shows that this phage has an evolutionary history involving FelixO1-related viruses but is in itself highly distinct from this group.

## Introduction

*Pseudomonas putida* strains are undoubtedly amongst the most ubiquitous and metabolically diverse bacteria on the planet, receiving substantial interest with respect to their bioremediation potential and in the involvement of various plant diseases [1, 2].

*P*. *putida* bacteriophages reflect the heterogeneity observed in their host, but have received little focus in comparison to phages infecting other Pseudomonads due to obvious clinical, and economic impacts other *Pseudomonas* species have upon humans, and our desire to utilise phages in the control of these. The heterogeneity of the Pseudomonads however, is reflected in their phages and this makes them an ideal system for understanding phage biology as a whole.

The first documented *P*. *putida* phage was gh-1 [3] which was found to exhibit morphological features typical of the *Podoviridae* family. Sequencing and analysis of this phage subsequently placed it within the T7-like group [4]. A study by Shaburova *et al*. [5] looked at the remarkable biofilm degradative activities of a number of *P*. *putida* phages, including pf16. Subsequent work deduced that the biofilm degradation exhibited by the *T7virus* phi15 and *epilson15virus* AF were due to virion associated factors [6, 7]. Aside from these, the only other *P*. *putida* phage research carried out was on the *Luz24virus* tf [8]. Localised nicks were identified and characterised in the tf genome and the associated consensus (5’-TACT*RTGMC-3’) was later shown to be present in related phages infecting *Pseudomonas fluorescens* and *Pseudomonas aeruginosa* [9, 10]. Additionally, this phage was shown to exhibit unique genome ends: a blunt right end and 4 nucleotide 3' overhang at the left end [8].

Here, we present the characterisation of two novel phages (pf16 and phiPMW) infecting *P*. *putida* PpG1. phiPMW was isolated from soil samples taken under a sycamore tree in the Midway Plaisance region of Chicago in 1977 by V. Krylov. Since then, its biological parameters have been determined and it has been utilised in a number of publications [5, 11, 12]. We demonstrate that phiPMW is a novel bacteriophage albeit with distant evolutionary relatedness to the “Felixounavirinae” [13].

The isolation of pf16 has an even longer history [14] and like phiPMW, several publications have arisen utilising this phage [5, 11, 15]. Both phages however, have remained uncharacterised at the genome level. We demonstrate that pf16 represents a peripherally related T4-like phage and show its evolutionary standing amongst the currently poorly classified T4 supergroup (*Tevenvirinae)*.

## Materials and methods

### Phage isolation and purification

Cultures of *P*. *putida* PpG1 (OD600 = 0.3) were inoculated at a MOI = 0.01. Chloroform was added immediately after culture lysis (~4 hours) to maximise yield after insensitivity was determined. The lysate was concentrated by PEG precipitation, and purified using CsCl density gradient centrifugation as described by Sambrook and Russell [16]. Bands were extracted and dialysis ensued with modified SM buffer (100 mM NaCl, 50 mM Tris, 10 mM, 8 mM MgSO4, 10 mM CaCl2) using Amicon Ultra-15 100kDa filters. DNA extraction, phenol-chloroform extraction, and ethanol precipitation, was carried out using SDS and proteinase K as described previously [16]. DNA was purified twice prior to sequencing using the MoBio DNA Purification Kit according to the manufacturer’s instructions.

### Electron microscopy

Carbon-coated formovar grids were subjected to hydrophilic treatment with poly-l-lysine for 5 min. Upon drying, CsCl purified and dialysed phage suspensions were deposited on the grids and successively stained with 2% uranyl acetate (pH 4.5). Samples were observed using a Phillips CM100 transmission electron microscope at 100 KeV.

### Library preparation and sequencing

Sequencing libraries were prepared from 50 ng of phage genomic DNA using the Nextera DNA Sample Preparation Kit (Illumina, USA) at the University of Cambridge Sequencing Facility. A 1% PhiX v3 library spike-in was used as a quality control for cluster generation and sequencing. Sequencing of the resulting library was carried out from both ends (2x300 bp) with the 600-cycle MiSeq Reagent Kit v3 on MiSeq (Illumina, USA) and the adapters trimmed from the resulting reads at the facility.

### Genome assembly and annotation

A total of 1,928,783 300 bp paired-end reads was obtained and underwent initial quality checking using FastQC [17]. Given the massive read coverage, stringent quality trimming parameters were utilised. Reads with a Q-score < 20, containing N’s, and with a final length < 40bp were discarded using Trimmomatic v0.32 [18].

Assembly was carried out using Geneious R8 (Biomatters, New Zealand), due to the difficulty other assemblers have in resolving terminal repetition often seen in phages. Geneious assemblies were checked for potential misassembly by comparison with output from SPAdes v3.6.2 [19] Genome length contigs were input to the scaffolding tool SSPACE to try to extend these in order to ensure the resolution of genome ends [20]. In addition, the two sequences obtained were utilised as references for read mapping using bbmap v35.x [21] and subsequent manual inspection in IGV to check for potentially ambiguous regions [22]. Pf16 was assembled with an average per base coverage of 4434x and phiPMW with 2849x.

ORFs were classified using the intrinsic Artemis ORF classification and GeneMark HmmS followed by manual verification of ribosomal binding sites [23]. Annotation was carried out using BLASTp, psiBLAST, and Hmmer algorithms, as well as enhanced domain classification using the Delta-BLAST algorithm [24]. Hmmer v3.1 was used to perform local hidden Markov model searches using custom databases [25]. Transmembrane domains and signal peptide sequences were identified using TM-Pred and SignalP respectively [26]. Coiled-coils were characterised using the Robinson algorithm implemented through STRAP (Software Tool for Rapid Annotation of Proteins) [27]. Cellular localisation of proteins was predicted by PSORTb [28]. tRNAs and were classified using tRNA-scanSE and Aragorn, and rho-independent terminators were identified using Arnold with an energy cutoff of -10 employed, following by manual inspection of the output from subsequent Mfold analysis and adjustment as necessary [29–32]. Frequency of codons recognised by tRNAs was carried out using an in-house script across both the whole genome and at the gene level.

To identify putative regulatory sequences, 100 bp upstream of every ORF in pf16 and phiPMW was extracted using an in-house script and analysed using both neural network promoter prediction for prokaryotic sigma 70 promoters and Multiple EM for Motif Elicitation to identify phage specific promoters [33]. Promoters were confirmed by using an additional in-house script on the extracted sequences which also provided confirmation as to the associated consensus.

### Protein bioinformatics

Molecular models were constructed from amino acid sequences using the I-Tasser package and energy minimisation of the first, most favourable prediction carried out on the Yasara server [34, 35]. Fidelity of models was assessed using the validation tools of the Whatif server before subsequent analyses. MetaPPISP was utilised to predict residues likely to be involved in protein-protein interactions [36]. Flexible residue driven docking then was carried out using HADDOCK with MetaPPISP predictions employed as directly interacting residues [37, 38]. At each docking stage energy minimisation and validation was carried out in an iterative approach to ensure the fidelity of models. Models were viewed and aligned using the PyMol package [39]. Quantitative similarity and structural superimposition between molecular models was provided with the TM-align script implemented through PyMol [40].

### Phylogenetic analysis

Concatenation of three typically conserved phage genes: the major capsid protein, terminase large subunit, and the replicative helicase was carried out followed by sequence alignments using Clustal Omega, T-Coffee, MUSCLE, and MAFFT [41–45]. T-Coffee was then used to generate a consensus alignment from information provided by the separate programs. Such an approach typically leads to more accurate alignments. The inference of phylogenies was then carried out using a Markov Chain Monte Carlo Bayesian approach implemented through the MrBayes package with sufficient generations to produce an average standard deviation of split frequencies for both pf16 and phiPMW of under 0.01 [46].

Roary was utilised in pan-genomic analyses with relevant plots produced using R [47].

BLAST based core gene analysis was carried out as follows: BLASTp searches of pf16 genes against T4 were carried out (E-value cutoff = 0.001) and plotted in a network style using Gephi for the purposes of clarity. Subsequently, the genes of all other *Tevenvirinae* candidates were compared to those of T4 (E-value cutoff = 1E-05) with matches extracted and built into a new database against which pf16 genes were searched (E-value cutoff = 0.001, Max hits per query = 1) in order to attempt to find additional T4-like gene matches.

## Results and discussion

### Biological characteristics

pf16 and phiPMW both infect *P*. *putida* PpG1, yielding clear plaques ~1 mm in size. Pf16 plaques however, are surrounded by a halo averaging 2 mm in size outwards from the plaque edge. The production of such halos is indicative of biofilm degradation by a phage [15, 48–52]. Both phages showed a complete absence of growth on the KT2440, UV4, 39D, and Ca3 strains of *P*. *putida* thereby hinting at a high level of specificity.

**Fig 1:**
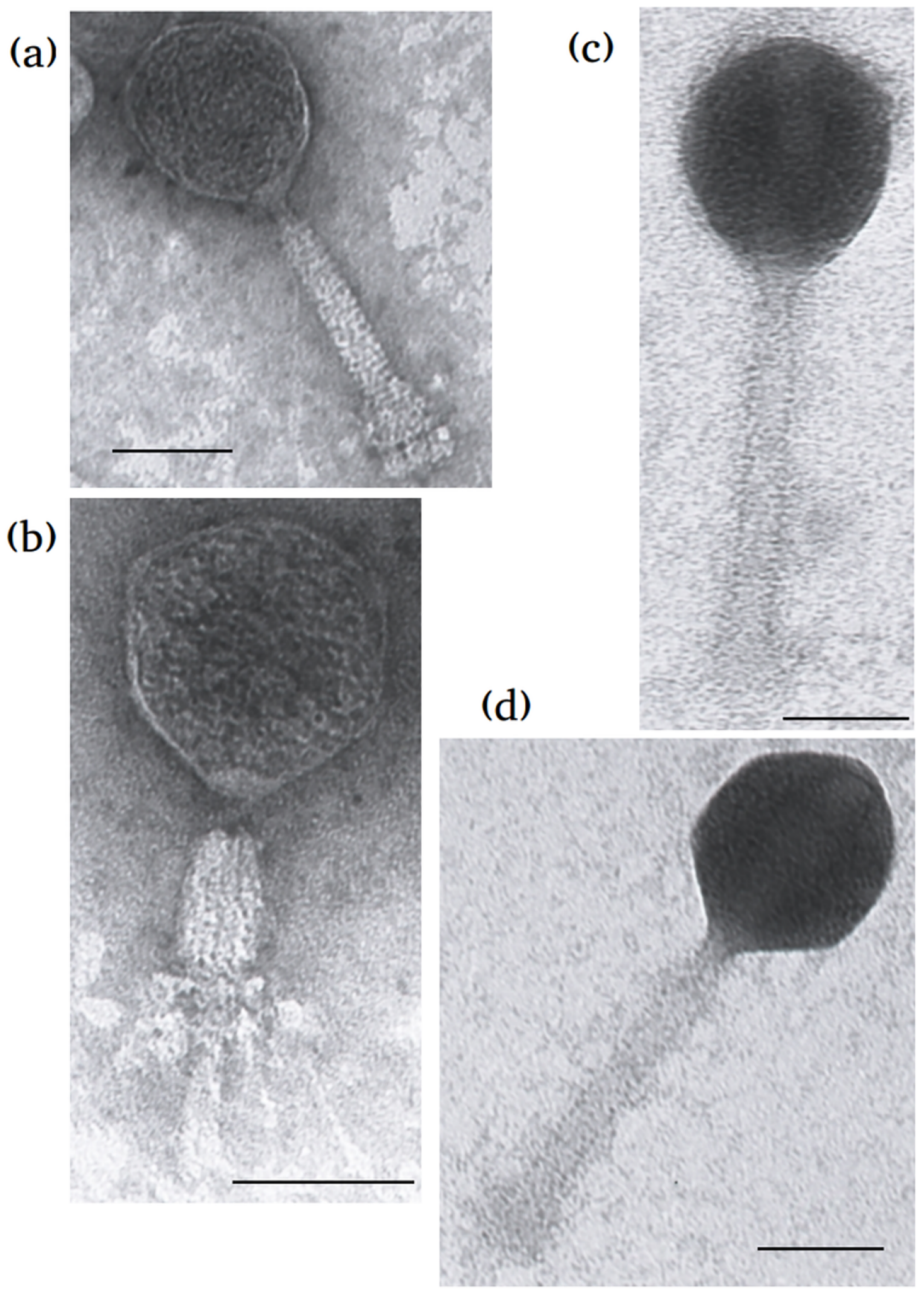
Electron microscopic images of (a) and (b), bacteriophage pf16 in non-contracted and contracted forms respectively, and (c) and (d) bacteriophage phiPMW. Scale bar corresponds to 100 nm.

Electron microscopic examination of pf16 (Fig 1) revealed a long contractile tail implicating it as a member of the *Myoviridae* family (order *Caudovirales)*. phiPMW tails also somewhat resemble those of the *Myoviridae* family (Fig 1), which is confirmed by subsequent genomic analysis. pf16 in particular held a slight resemblance to classic images taken of *Enterobacteria* phage T4. The capsid length and width of pf16 is approximately 88 nm (+-0.39 nm) x 82 nm (+- 0.38 nm) with a tail length and width of approximately 148 nm (+- 0.45 nm) x 20 nm (+- 0.33 nm). phiPMW proved to be the larger of the two with capsid dimensions of 107 nm (+- 0.51 nm) x 93 nm (+- 0.47 nm) and 166 nm (+- 0.47 nm) x 19 (+- 0.28 nm) nm for the tail.

### Genomic features

#### General features of the pf16 and phiPMW genomes

Restriction analysis of both pf16 and phiPMW with PvuII, EcoRV, BamHI, HindII, and DraI, resulted in a very small level of digestion for pf16 for DraI only (data not presented). The DraI recognition site is AAATTT whilst all others contain at least one C and/or G. All sites are present in pf16 to various frequencies suggesting the presence of modified cytosine and/or guanine bases. With respect to phiPMW, digestion was achieved with all enzymes apart from PvuII for which it lacks sites (data not presented).

**Fig 2:**
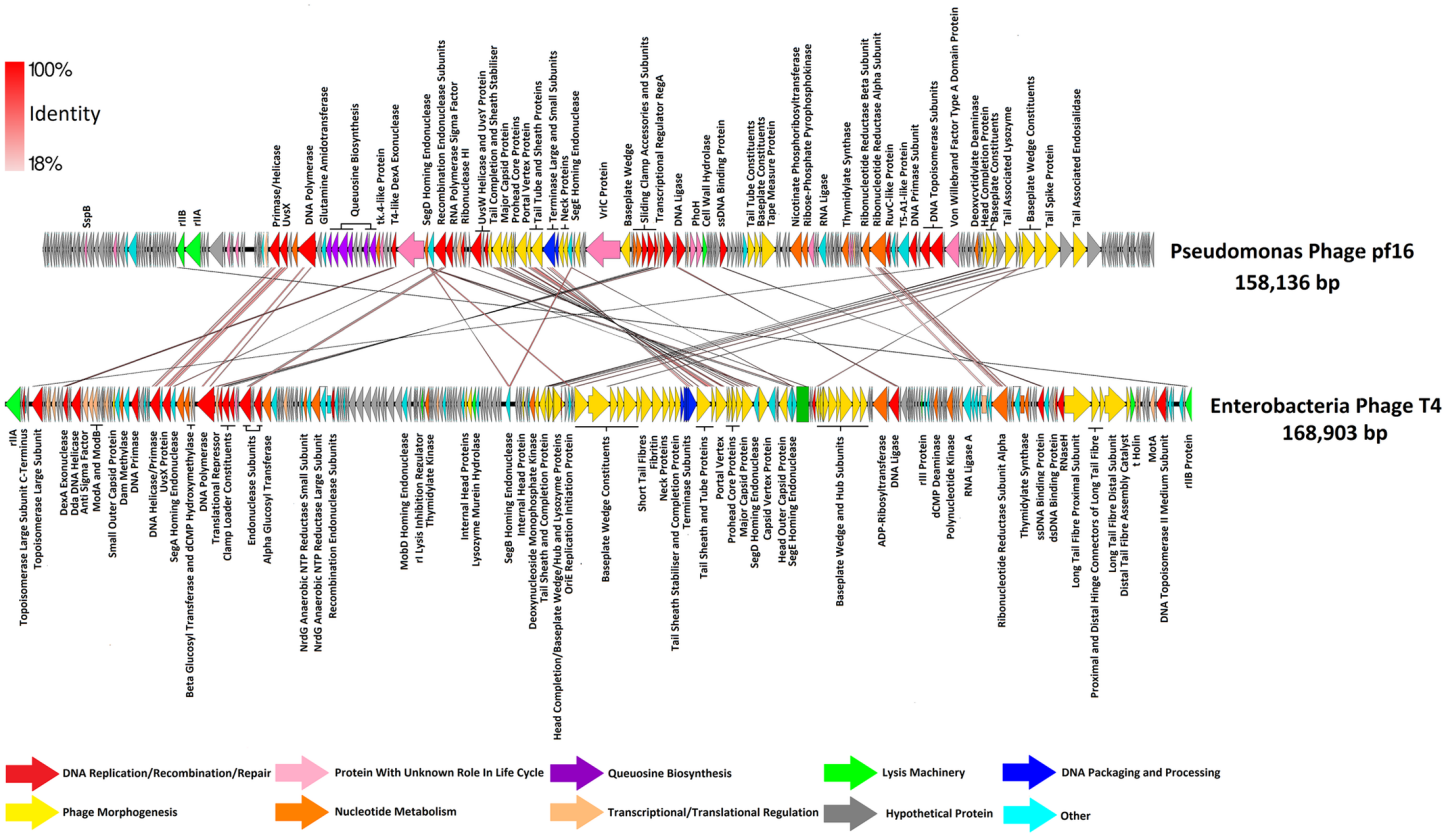
Comparative genome composition of the *Pseudomonas putida* phage pf16 and *Enterobacteria* phage T4. Predicted ORFs are presented as arrows indicating the direction of transcription. Arrows are coloured by function according to the key presented at the bottom of the figure. Functional annotations (if any) are given alongside respective ORFs. Red shading between phages indicates percentage amino acid identity according to the key given.

**Fig 3:**
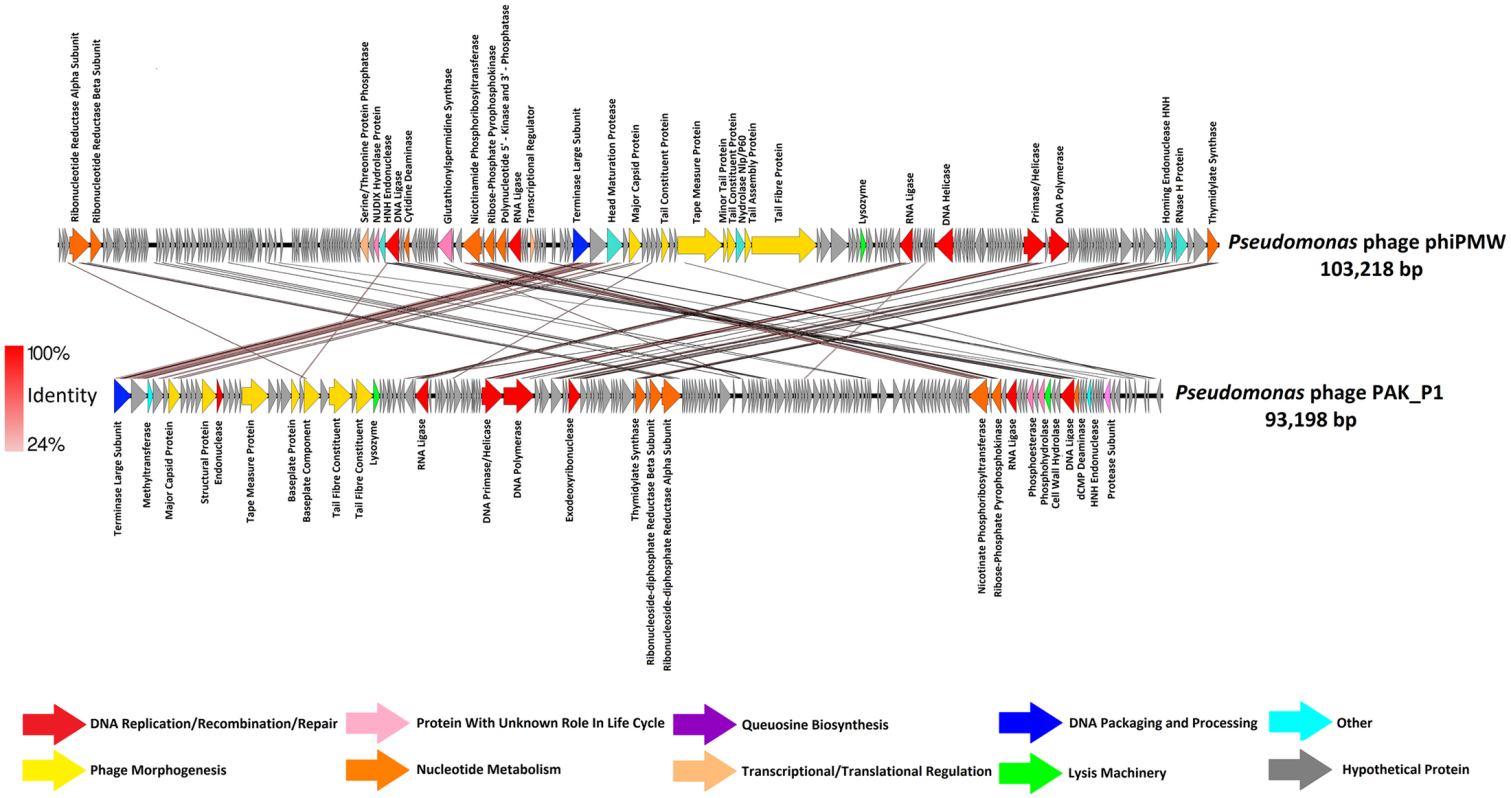
Comparative genome composition of the *Pseudomonas putida* phage phiPMW and *Pseudomonas aeruginosa* phage PAK_P1. Predicted ORFs are presented as arrows indicating the direction of transcription. Arrows are coloured by function according to the key presented at the bottom of the figure. Functional annotations (if any) are given alongside respective ORFs. Red shading between phages indicates percentage amino acid identity according to the key given.

pf16 and phiPMW possess dsDNA genomes of 158,136 bp and 103,218 bp of which 93.3% and 93.4% (respectively) has coding potential. A circular assembly representation for pf16 was produced, suggesting circular permutation typical of T4-like phages. The GC content of the pf16 genome is 52.65%, whilst phiPMW has a lower value of 45.15%, both significantly lower than the 61.5% and 62.3% observed in *P*. *putida* reference strains KT2440 and NBRC 14164 (NC_002947.4, NC_021505.1). GC skew minima in genomes, a strong oriC indicator [53], revealed putative replication origins at 32,001 bp and 40,001 bp for pf16 and phiPMW respectively. 237 ORFs were predicted for pf16 and 229 for phiPMW. In the case of pf16, 217 ORFs use ATG, 18 use GTG, and 3 use TTG as start codons. With respect to start codons in phiPMW, 199 ORFs use ATG, 21 use GTG, and 9 use TTG. Transcription of these ORFs occurs on both strands in both phages. Genome maps of pf16 and phiPMW are presented in Figs 2 and 3 with more in-depth ORF analysis presented in S1 and S2 Tables for pf16 and phiPMW respectively.

In the pf16 genome, 18 ORFs were identified as being associated with processes involved in DNA replication, recombination, and repair. 15 ORFs carry out various aspects of nucleotide metabolism, 23 proteins were identified as structural constituents, and 2 encode the terminase small (TerS) and large subunits (TerL). Hidden Markov models (HMMs) were unable to detect any analogues of dCMP hydroxymethylase enzymes, suggesting that pf16 does not share the hydroxymethylcytosine bases found in phage T4 [54]. However, 6 ORFs comprise a module of genes directing the metabolism of queuosine; a modified guanosine derivative. Amino acid homology indicates that this module was derived from vibriophages and is speculated as being a method of increasing the pool of phage specific queuosine-containing tRNAs, allowing protein synthesis to be shunted in its favour [55]. The downstream locality of a Vibriophage-related SegD homing endonuclease was likely involved in the acquisition and dissemination of this cluster.

Pf16 contains two peptidoglycan-degrading genes. One is a tail-associated lysozyme similar to the gp5 baseplate hub subunit from enterobacteria phage T4. The putative endolysin involved in host cell lysis however, contains Hydrolase_2 domains found extensively in *Bacillus* species, and which play a role in sporulation [56].

**Fig 4:**
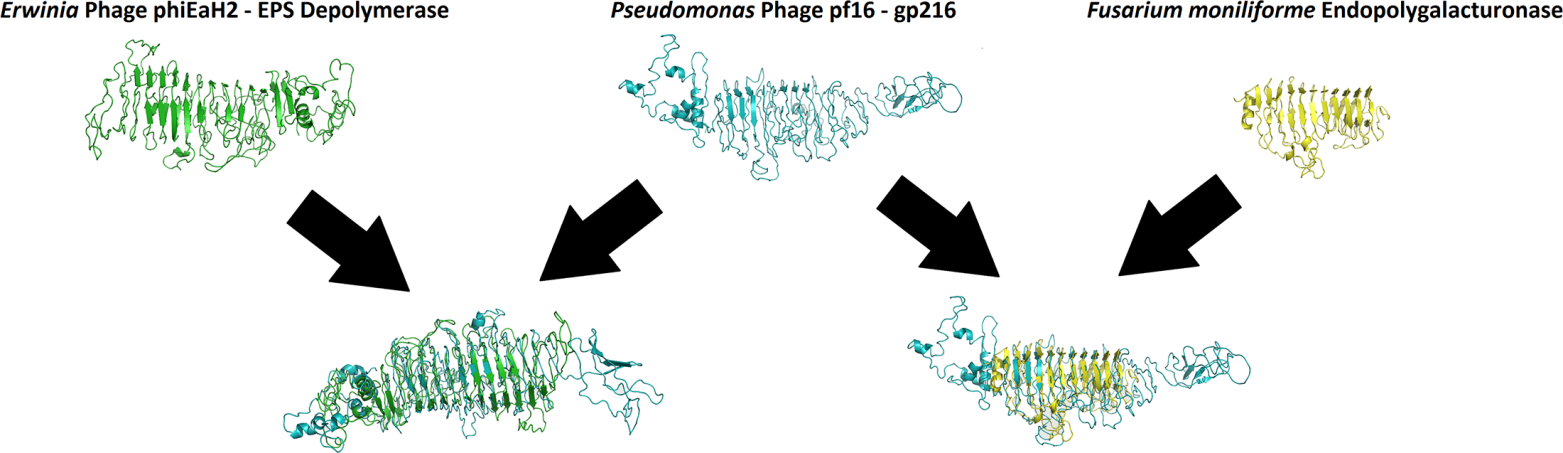
Molecular models and superimposition of EPS-depolymerase like enzymes from *Pseudomonas* phage pf16, *Erwinia* phage phiEaH2, and *Fusarium moniliforme*. Pf16 is shown in blue, phiEaH2 in green, and the fungus *Fusarium moniliforme* in yellow.

As far as identifying a diffusible component responsible for biofilm degradation, uncertainty abounds. Currently, there are 9 different enzyme classifications for proteins involved in the degradation of extracellular polymeric substances (EPS) [57]. These are: sialidase, levanase, xylosidase, dextranase, alginate lyase, pectate/pectin lyase, hyaluronate lyase, lipase, and peptidase activities. *Myoviridae* have thus far been associated with EPS depolymerases exhibiting sialidase, pectate/pectin lyase, and peptidase activities [57]. Gp215 of pf16 is a putative tail-associated endosialidase likely involved in capsular degradation as opposed to biofilm destruction. Gp214 and gp216 both contain low E-value domains (Pectate_lyase_3 (E-value = 1.42e-05) and Beta_helix (E-value = 1.01e-03) in gp214, and Beta_helix (E-value = 4.44e-04) in gp216) found in pectate lyase enzymes and so one or both of these may play a role in biofilm breakdown. Gp216, specifically, was the only protein in pf16 with predicted localisation within the extracellular domain (S1 Table). *Ab initio* molecular modelling was carried out on this, along with an EPS depolymerase from *Erwinia* phage phiEaH2 [58]. Surprisingly, in the absence of detectable sequence homology, the two proteins share a similar elongated fold containing a significant quantity of residues engaged in β-sheets, with a TM-align similarity score of 0.5861 (>0.5 indicates that both proteins share the same fold) (Fig 4). A subsequent 3D blast of the pf16 gp216 model against the PDB database yielded an endopolygalacturonase from the phytopathogenic fungus *Fusarium moniliforme* as the most significant hit (E-value = 2e-29)[59]. A structural alignment of this and gp216 is presented in Fig 4. Structural similarity of such proteins has been previously reported for the prokaryotic domain [60]. Endopolygalacturonase, also known as pectin depolymerase, is a monomeric protein that hydrolyses the alpha 1,4 glycosidic bonds between galacturonic acid residues, which are the major constituent of pectin [61]. Taken together, this all points towards gp216 being the most probable EPS depolymerase candidate.

Amongst other ORFs, 146 of the predicted genes within pf16 encode hypothetical proteins to which no putative functions have been ascribed.

The genome of phiPMW is much more enigmatic than that of pf16. 198 of the 229 ORFs predicted have no ascribed functionality and 139 have no homology whatsoever within the databases at the amino acid level, highlighting truly remarkable novelty. Amongst the 31 known ORFs, 5 of these have functions involved in DNA replication, recombination, and repair. Seven are involved in various aspects of nucleotide metabolism, 9 are products with structural and DNA packaging roles, and gp150 encodes a putative glycoside hydrolase type lysozyme. By location, either gp149 or gp151 could be the putative holin or spanin complex due to the presence of transmembrane domains in both. An unusual gene predicted in phiPMW is a putative glutathionylspermidine synthase (gp102). The function of this enzyme is unclear but it is also encoded in the genomes of *Pseudomonas* phage Lu11 [62] and *Deftia* phage phiW-14 (NC_013697).

#### Regulation of gene expression in pf16 and phiPMW

pf16 contains 84 Rho-independent terminators [63] whilst phiPMW has 40. A number of these terminators exist as bifunctional units (17 in pf16 and 6 in phiPMW) whereby two such elements face one another on both strands in both phages. This reflects the partially palindromic nature of these elements. Some of these terminators however, lie on apparently non-coding regions of the opposite strands. Despite this, we cannot dismiss these as functionless, as they may regulate or form part of some undetected ORF, or act as downstream terminators to prevent read through of transcripts. pf16 has a bidirectional terminator at every point whereby transcription switches from one strand to the next, thereby providing a clear division between the different transcription units and preventing readthrough. This type of regulation is reminiscent of the *Luz24virus* genus of phages which possess two such units split by one of these terminators [10, 64]. None of the bidirectional terminators present in phiPMW divide specific transcription units on both strands.

With respect to promoters utilised by phiPMW, genes are found to be largely under the control of sigma70 promoters, with 30 likely candidates identified within the genome (Table 1). For pf16, 15 sigma70 promoters were detected (Table 2). The low number of sigma70 promoters in pf16, in comparison to its genome size, is explained by the presence of phage specific promoter elements. Analysis revealed 56 instances of a specific promoter conforming to a consensus of 5’–CATCAACACAGTCAACAACGAATACATC– 3’. A representation of putative pf16 promoters is given in S1 Fig. This finding is not overly surprising, due to the presence of gp98, a putative RNA polymerase sigma factor for transcription of late genes. This is a feature well documented in T4 [65]. In addition, 13 instances of a weaker motif (E-value = 5.9e-012, consensus: 5’–ACAAAAAGGGCTCCTTGGAGGGCTTTTCTTTGA– 3’) were also predicted. This may point towards a system of early, middle, and late transcription for which T4 is well known [65]. In T4, there are 50 late promoters conforming to a consensus of TATAAATA, which control the expression of genes involved in head and tail morphogenesis [65]. Three perfect matches to this motif were discovered within pf16 in intergenic regions lying upstream of gp106, gp124, and gp125, encoding the major capsid protein (MCP), VrlC-like protein (most probably a structural component) and a baseplate wedge subunit respectively. These are all genes expected to be under the expression of late promoters. Interestingly, Gp69 and gp131 of pf16 encode DsbA and RegA equivalents, known in T4 to enhance late gene expression and suppress the translation of early gene mRNAs through competitive ribosomal binding respectively [65, 66].

**Table 1:** Table showing sigma70 promoter elements within *Pseudomonas* phage pf16.

Start	End	Strand	Promoters	Gene Immediately Downstream
14925	14954	-	TTGACACGCCTGTGCTATGGCAGTAATATA	Gp35 –Hypothetical Protein
16392	16421	-	TTGACACCTTACCTTGCACGTCGTACTATA	Gp43 –Hypothetical Protein
25660	25688	-	TTGACACCAACCAACACCTTCTGTAAATT	Gp55 –Hypothetical Protein
39647	39673	-	TTGACATCATTACCCTGGACCTATAAA	Gp75 –Hypothetical Protein
44762	44790	-	TTGACACAGGTAGTGGTAGCACTTAAACT	Gp81 –Hypothetical Protein
60076	60104	+	TTGACACTCATACCTAACACCACTACACT	Gp99 –Hypothetical Protein
85240	85267	+	TTGACAAGTAAGACAAACCGTTTAAACT	Gp127 –Sliding Clamp DNA Polymerase Accessory Protein
86054	86076	+	TTGACAGGCACCCTTTCCTTATTAT	Gp128 –Clamp Loader Subunit I
113641	113667	+	TTGACACGGGTGTCACATTACCATAAT	Gp174 –Hypothetical Protein
139141	139169	-	TTGACACCTACCCTACCTTATCATATTAT	Gp49 –Hypothetical Protein
135729	135757	-	TTGACAAGCTCAGGCAAGACAAGTTTAAT	Gp202 –Hypothetical Protein
146035	146064	-	TTGTGATCCTTGTGGTGGCTGTACTATAAT	Gp29 –Antirestriction Protein
146681	146708	-	TTGACATTGCATGCCCTTCTATGATAAT	None
157254	157282	-	TTGACACTTACCATTGATAGTCATACAAT	Gp2 –Hypothetical Protein
157721	157749	-	TTTACACCTTGTGTGGTGAGGTGTATAAT	None
**Promoter Consensus:** **TTGACA**CCNNCCTTNNNCNTNCTCA**TAAAAT**				

**Table 2:** Table showing sigma70 promoter elements within *Pseudomonas* phage phiPMW.

Start	End	Strand	Promoters	Gene Immediately Downstream
3684	3713	+	TTGACAACCGCTGAGGCAAAGGATTATATT	Gp6 –Hypothetical Protein
3964	3991	+	TTGACACCACCATCAGAGGTGATAGAAT	Gp6 –Hypothetical Protein
5997	6026	+	TTGACAAATTTGATCGTCAAAACCTGTTCT	Gp12 –Hypothetical Protein
9809	9837	+	GTGACATGGGTATAACATCCACGTATAAT	Gp25 –Hypothetical Protein
10425	10453	+	TTGACAACGTTAGCAGCTTTGATTAGAAT	Gp26 –Hypothetical Protein
11448	11475	+	TTGACACCCACTGTAAACCCTGTAAAAT	Gp30 –Hypothetical Protein
11838	11866	+	TTGACAAGGTTAGCACCTAATGACATAAT	Gp31 –Hypothetical Protein
12401	12428	+	TTGACAAGGATGCCAGTTGTGCTAATCT	Gp32 –Hypothetical Protein
15129	15156	+	TTGACAAGGTAAACGGATCCTGTAGAAT	Gp41 –Hypothetical Protein
16112	16140	+	TTGACACACACCCTGATCATGCGTATTAT	Gp45 –Hypothetical Protein
17111	17138	+	TTTACAACGTTTTCAGTTGGCGTATAAT	Gp51 –Hypothetical Protein
17712	17739	+	TTGACACCTTACCCTAAAGTGATAGAAT	Gp53 –Hypothetical Protein
18378	18405	+	TTGACCTGTTCTGTGATCGGCGTATAAT	Gp55 –Hypothetical Protein
25695	25723	-	TTGACAACCATATCAAAGTATTCTATAAG	Gp80 –Hypothetical Protein
26030	26055	-	TTGACAGTATTCGACAGGCTTGTAAG	Gp81 –Hypothetical Protein
26108	26137	-	TTGACACACTACTACTATTATGGGTATAGT	Gp81 –Hypothetical Protein
38558	38586	-	TTGACAGGTTACTCCATGAACGATATGAT	Gp106 –Hypothetical Protein
41794	41821	-	TTGACAAGGGTTTTCCCTCGTGTAGAAT	Gp114 –Hypothetical Protein
42350	42377	+	TTGACACAACCCTAGCACCTGATACAAT	Gp116 –Hypothetical Protein
44591	44620	+	TTGACAAATATCACAAGTAGTTGGTAATAT	Gp123 –Hypothetical Protein
45738	45767	+	TTGACAAACATGGGGGCTTTCCTACATAAT	Gp126 –Terminase Large Subunit
48451	48479	-	TTGACAAGTTTGACAAGCTGGTCTTTAAT	None
57659	57688	+	TTGACACTCGTGAACTCACTGAAGTAAAAT	Gp139 –Minor Tail Protein
59063	59092	+	TTGACAAATTGGAATTCTACATAGTAGAAT	Gp139 –Minor Tail Protein
64204	64233	+	TTGACAAAGCACTGTATGAGCGTCTAGAAT	Gp144 –Hypothetical Protein
82244	82271	-	TTGACATGCCACCAGAACCCTGTAGAAT	Gp180 –Hypothetical Protein
82304	82333	+	TTGACAGGAAGCTCCTCGGTTTCCTATACT	Gp184 –Hypothetical Protein
83284	83312	+	TTGACAGGGACTACGAATCAGGATATATT	Gp185 –Hypothetical Protein
83538	83562	+	TTGACAACGTTCTGATCGGTAAAAA	Gp186 –Hypothetical Protein
102121	102145	+	TTTACAAAGGAACGGGAGCTATAAT	Gp229 –Thymidylate Synthase
**Promoter Consensus**: **TTGACA**AGGTTNTCAAATCTTGTA**TATAAT**				

When phiPMW was subjected to the same analysis, no phage specific promoters were found. When repeated ahead of each putative operon, 9 weak promoter-like candidates were indicated with a consensus of 5’-TAGTTTCNNATATCAANAGANNCTTG-3’ (S1 Fig). Positions 14, 15, and 26 (S1 Fig) are completely conserved amongst all elements with no instances of overlap with previously described sigma70 promoters. However, no sigma factors or RNA polymerase/polymerase-modifying enzymes have been identified capable of recognising these elements. Therefore, it cannot be stated with confidence whether these are active promoters *per se*.

#### Analysis of tRNA genes and codon usage

A fundamental method to control gene expression levels within a virus is through codon usage adaptation to more closely match that observed within its host. Almost half a century ago T4 phages were found to carry tRNA genes [67], the deletion of which decreased infection productivity [68]. Previous reports suggested multiple explanations as to the presence of tRNAs within phages including their usage as a point of integration into host genomes [69, 70], however this fails to explain their presence within non-temperate phages. It has also been reported that phage tRNA genes are more selective for abundant codons within the phage which are rarely found within the host [71]. The role these genes may play within pf16 and phiPMW was therefore investigated.

Like T4, pf16 contains 8 tRNAs (Tyr, Met, Asn, Pro, Lys, Thr, Trp, and Ser) present in three distinct clusters and adjacent to gp65, a YqeY motif-containing protein speculated to play a role in tRNA charging [72], in contrast to T4’s two clusters. The clusters in T4 are separated by a SegB homing endonuclease which may permit enhanced horizontal gene transfer of these genes [65]. Such an element is absent in pf16.

phiPMW has 6 tRNAs (Ile, Phe, Asn, Trp, Thr, and Pro) in two distinct clusters, differing from the “Felixounaviruses” infecting *Pseudomonas* species, which for *P*. *aeruginosa* contain either 3 or 11–13 tRNAs [13]. The recently characterised KIL-related viruses of *P*. *syringae* phages within the “Felixounavirinae” [73] possess either 5 or 9 tRNAs thus far, showing greater similarity to phiPMW.

S3 Table shows codon usage patterns across pf16 and phiPMW, highlighting those codons recognised by phage-encoded tRNAs and their position relative to codon usage by *P*. *putida*. 4 of the pf16 tRNAs recognise the top 12 most abundant codons in the pf16 genome whilst all six phiPMW tRNAs recognise codons within the top 50% used by the phage. 4 of the codons recognised by pf16 and phiPMW tRNAs are also in the bottom 50% of codons utilised by *P*. *putida*, showing a bias towards codons prevalent in the phages and rarer in the host, a result similar to a previous study [71]. We also decided to look at tRNA codon bias at the gene level to investigate whether phage tRNAs offer a distinct method of gene regulation. S2 Fig shows the ORFs with those codons recognised by pf16 and phiPMW tRNAs. Whilst a number of ORFs contain 20% or more codons recognised by phage tRNAs, none were confirmed as genes expected to be expressed at high levels in the replication cycle (e.g. structural proteins). It may be however, that these tRNAs have been recently acquired and thus significant codon usage biases have yet to develop.

#### Pf16 stringent starvation protein B

One particularly interesting finding within the pf16 genome is an ORF (gp18) encoding an analogue of the stringent starvation protein B (SspB)[74]. This is something yet to be reported in any other phage to date. In *E*. *coli*, SspB has been shown to play two roles. The first is the *ssrA* (tmRNA) response, essential in the resolution of stalled ribosomes [75, 76]. Using BLAST, HMMs constructed from the total tmRNA database [77], and sequence alignment/secondary structure analysis of relationship to known tmRNAs structures, found no evidence of an ssrA-like element within pf16 (Data not presented).

A secondary role of SspB in the cell is in the regulation of the sigma factor RpoE. Under non-stressed conditions, RpoE remains in an inactive state complexed with its cognate anti-sigma factor RseA. Under nutrient stress, upregulated SspB binds RseA tagging it for degradation by ClpXP, subsequently releasing RpoE which enacts the extracytoplasmic response [78].

**Fig 5:**
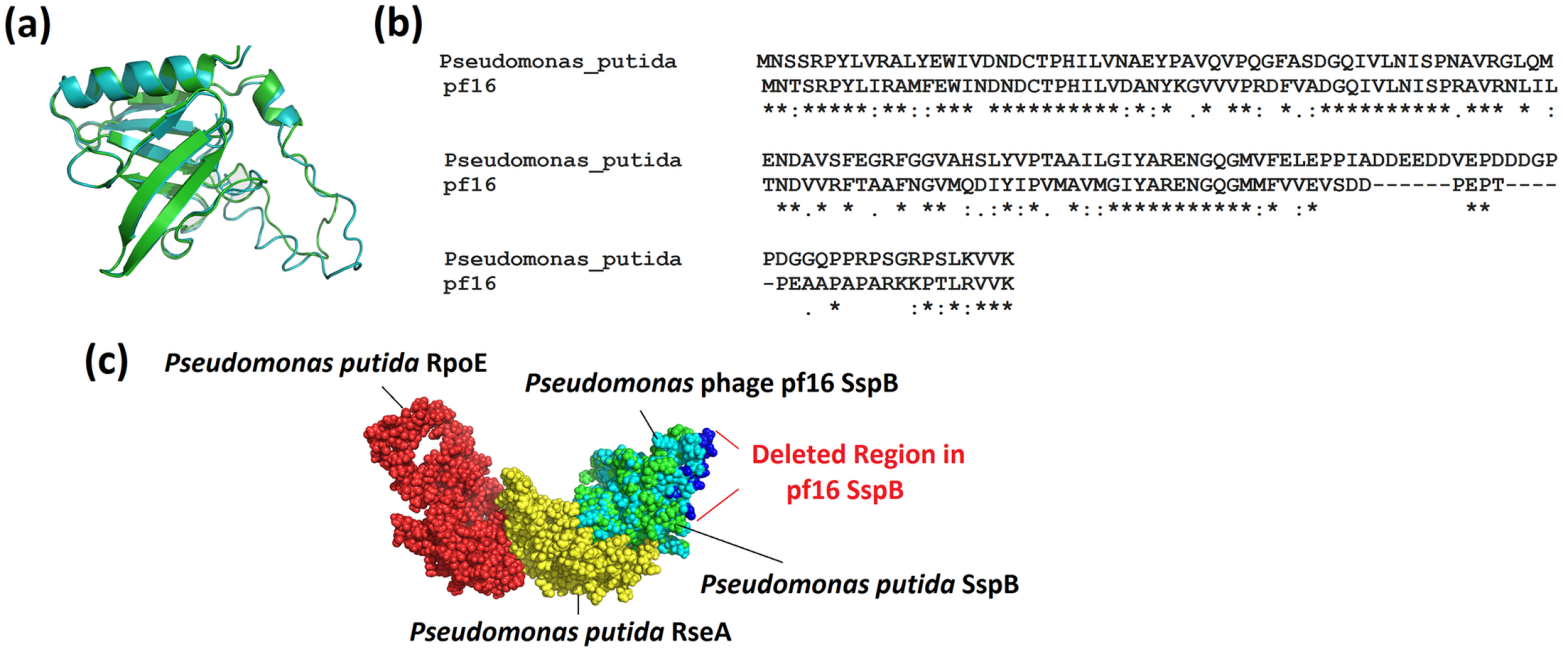
Analysis of *Pseudomonas* phage pf16 stringent starvation protein B (SspB). (a): Superimposition of *Pseudomonas putida* and pf16 SspB molecular models with resulting TM-align score of 0.818 indicating a high level of structural similarity. (b): Sequence alignment of *P*. *putida* and pf16 SspB proteins showing a high level of identity. Note the deletion in pf16 of numerous negatively charged residues (aspartate and glutamate). (c): Predicted complex between *P*. *putida* sigma factor RpoE, anti-sigma factor RseA, and both *P*. *putida* and pf16 SspB protein models highlighting the overlap in binding sites and thus putative competitive inhibitory action by pf16 SspB. The deletion in pf16 SspB compared to *P*. *putida* is highlighted in dark blue.

This was investigated utilising a molecular modelling approach. pf16 and *P*. *putida* SspB models were constructed, revealing remarkable structural similarities (Fig 5a) (TM-align score: 0.818). Sequence alignment of the two proteins revealed high levels of homology, the only significant difference being the deletion of a small region containing predominantly negatively charged residues (Fig 5b) lying within the most intrinsically disordered region of the *P*. *putida* SspB; regions often implicated in protein-protein interactions (data not presented). The loss of these residues is also associated with a drop in the disorder observed in that region in the pf16 SspB, suggesting a role for this as a site of interaction.

Residue driven docking of both SspB proteins was carried out to the *P*. *putida* RseA, previously docked to RpoE using MetaPPISP predicted interaction residues. It was found that both SspB proteins bind the same site on RseA; this overlap can be seen in Fig 5c. Additionally, the deleted region in pf16 compared to *P*. *putida* shows no overlap with the RseA binding site and is free to interact with other components, potentially including ClpXP. These similarities between the pf16 and *P*. *putida* SspB proteins suggest similar functionalities, with the absence of a putative binding site in pf16 highlighting a potentially key difference which merits experimental investigation.

#### Host tropism determinants

It is well documented that the tail fibre protein, involved in receptor adsorption, forms the major tropism determinant in phages [79, 80]. In both pf16 and phiPMW, the tail fibre was identified as gp213 and gp143 respectively. Given the intriguing phylogeny of pf16 discussed later, the tail fibre proteins of both phages were investigated to try and gain some insight into the nature of their host specificity.

The phiPMW tail fibre is a very large protein, 1922 amino acids in length and containing phage_tail_3 superfamily domain (E-value = 0.0), that shows homology to host specificity proteins found in *P*. *fluorescens* at 40% identity and 98% query cover (Accession: WP_063030542). Most variability lies within the C-terminal region; a pattern often observed with respect to differences in receptor utilisation [81]. Given the complete lack of homology with described phages, including the “Felixounavirinae”, phiPMW likely utilises a receptor distinct from those of known phages.

**Fig 6:**
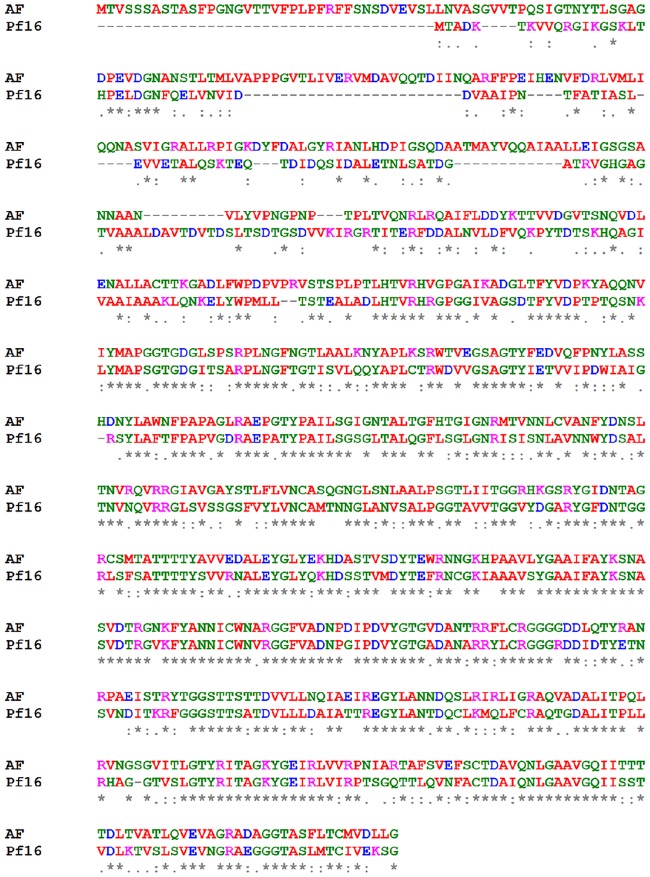
Sequence alignment of the *Pseudomonas* phage pf16 and *Pseudomonas* phage AF tail fibre proteins. Alignment of the pf16 and AF tail fibre proteins highlighting particular conservation of the C-terminal region.

The pf16 tail fibre protein held further interesting insights upon investigation. At 660 amino acids in length, this showed a 61% level of homology at 81% query cover to the tail fibre protein from phage *A*F [7]. AF, like pf16 and phiPMW, infects the PpG1 strain of *P*. *putida*. Upon sequence alignment, we find that the majority of conservation is confined to the C-terminal region of the two proteins, clearly implicating it as the region involved in receptor attachment (Fig 6). When we consider the phylogenetic traits of pf16 discussed below, the importance of the tail fibre protein in determining host tropism is evident.

### Evolutionary and Comparative Analyses of the *Tevenvirinae* and “Felixounavirinae”: Where do pf16 and phiPMW fit in?

#### Pf16

Despite the ubiquity and abundance of T4-like phages within the environment and the many hosts they infect (revealed by marker based studies), and decades of *Pseudomonas* phage research, it is quite enigmatic as to why no T4-related phages have been reported for this genus [82–84].

Of the 237 ORFs predicted within pf16, 62 have best hit homology to various T4-related phages, 14 of which are cyanophages. In addition, another 9 ORFs are homologous to MedDCM viral contigs derived from a study investigating viral constituents in the Mediterranean [85]. Many of these genes are reminiscent of putative T4-related marine phages. Indeed, gp139 encodes a putative *phoH* protein; one of several auxiliary metabolic genes (AMGs). AMGs encoding various phosphate regulon genes are widely reported constituents of marine phages though *phoH*, whose function remains largely unknown, is the most prevalent of these. Indeed, previous work suggested the use of the *phoH* gene as a means of classifying marine phages, though in pf16’s case this *phoH* shows an independent origin unrelated to other phages and cannot be used as a marker in this instance. Even though it is not present in all marine phage genomes, the independent acquisition of this gene implies an advantageous accessory role in the phage life cycle [86, 87].

**Fig 7:**
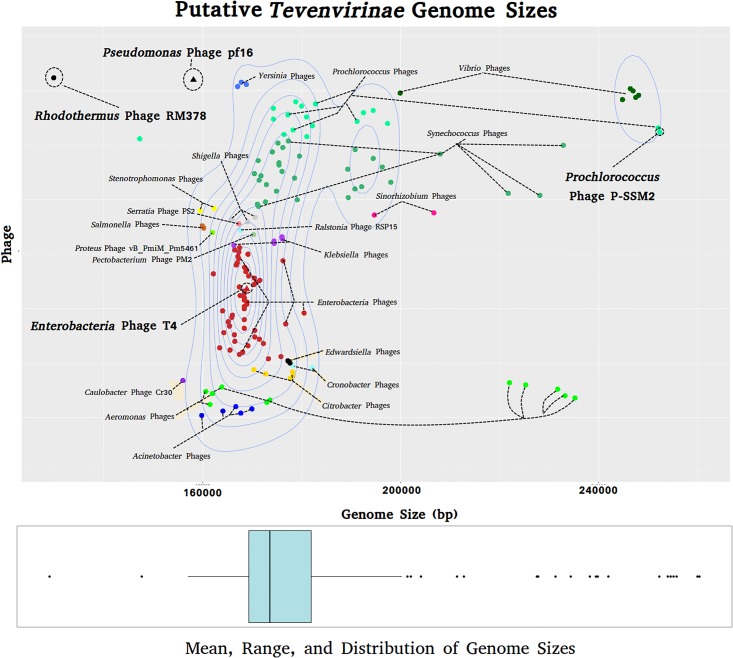
Graph showing distribution of putative *Tevenvirinae* genome sizes. Phages infecting related hosts are colour coded appropriately with labels provided specifying the phage or group of phages. *Pseudomonas* phage pf16, *Rhodothermus* phage RM378 (smallest genome), *Prochlorococcus* phage P-SSM2 (largest genome), and *Enterobacteria* phage T4 are circled and labelled in bold. Contour density lines shows clustering of most phages around similar genome sizes. Boxplot at the bottom of the figure summarises the distribution of the phages. The main box and associated lines shows the spread, mean, and quartiles of the main cluster observed within the major contour lines with outliers and the smallest/largest genomes represented as dots.

pf16 also encodes many genes observed within *Tevenvirinae* members, for example, the *rIIa* and *rIIb* lysis regulatory system, the *DsbA* transcriptional regulator, and the *DexA* exonuclease. Previous studies suggested that T4-related phages share a conserved set of core genes, the remainder constituting an “accessory genome” meeting the extra normal metabolic needs of each phage. This accessory genome contains various AMGs that evolve via the modular evolutionary paradigm. Arguably the most well known AMGs amongst T4-related phages are photosynthetic genes, as observed in the *Synechococcus* phage S-PM2 [88]. Such genes may play a profound role in the biogeochemical cycles which shape our planet. At the time of writing, we found 156 putative members of the *Tevenvirinae* within the databases. These candidates were obtained based on homology in conserved genes (major capsid protein, terminase large subunit etc) as well as possessing genes typically found within members of the *Tevenvirinae*. The genomes display an impressively variable size range (Fig 7), the smallest belonging to *Rhodothermus* phage RM378 at 129,908 bp and the largest being 252,401 bp and belonging to *Prochlorococcus* phage P-SSM2. This highlights the potential plasticity of these accessory regions. Interestingly, the largest T4-like phages are on average those infecting *Vibrio* species, followed by *Aeromonas* phages, and then the Cyanophages. Even though pf16 shows a potential cyanophage phylogenetic origin and has had past evolutionary interactions with large *Vibrio* T4-like phages (based upon the acquisition of the queuosine module), it possesses the fourth smallest genome, fitting into neither group (Fig 7).

**Fig 8:**
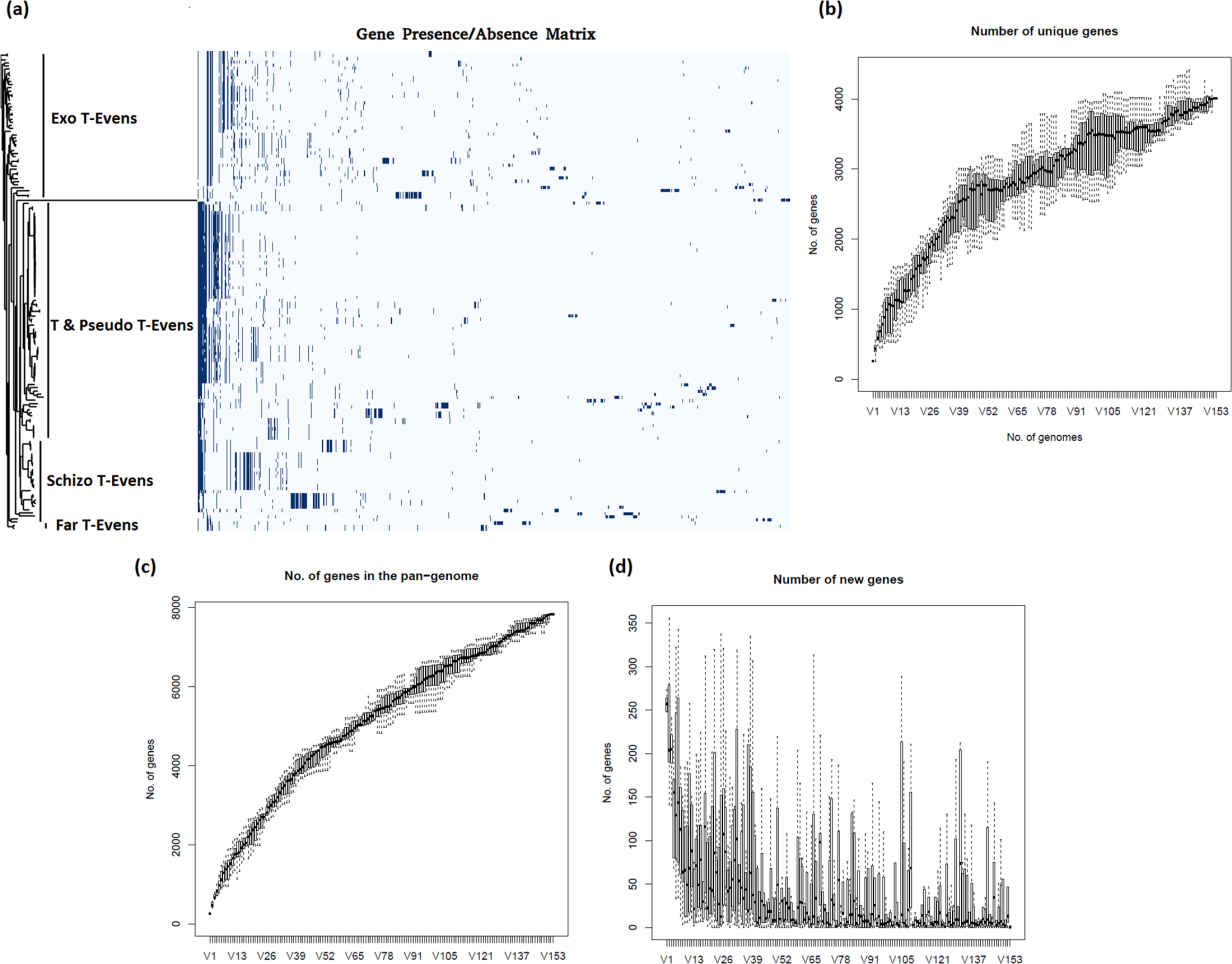
Pangenomic analyses of the *Tevenvirinae*. (a) Gene presence/absence matrix plot. Blue regions correspond to genes present in a minimum of two phages on the extreme right hand side and increasing to all phages (if applicable) on the extreme left. (b) A plot of unique genes against the number of genomes. Numbers of unique genes rises with genome number highlighting the fact that individual members of the *Tevenvirinae* contain a significant quantity of novel genes. (c) A plot of the number of pangenomic genes (those not common to any phages) against number of genomes showing a steady rise in the T4-like pangenome and highlighting the wide array of accessory genes present in this group. (d) Graph showing number of completely new genes against genome number in each successive phage highlighting that despite a steady increase in unique genes across the entire *Tevenvirinae*, the number of new genes with respect to each additional phage steadily declines showing that there is a limit on the novelty and that there is some commonality across a large majority of members.

**Fig 9:**
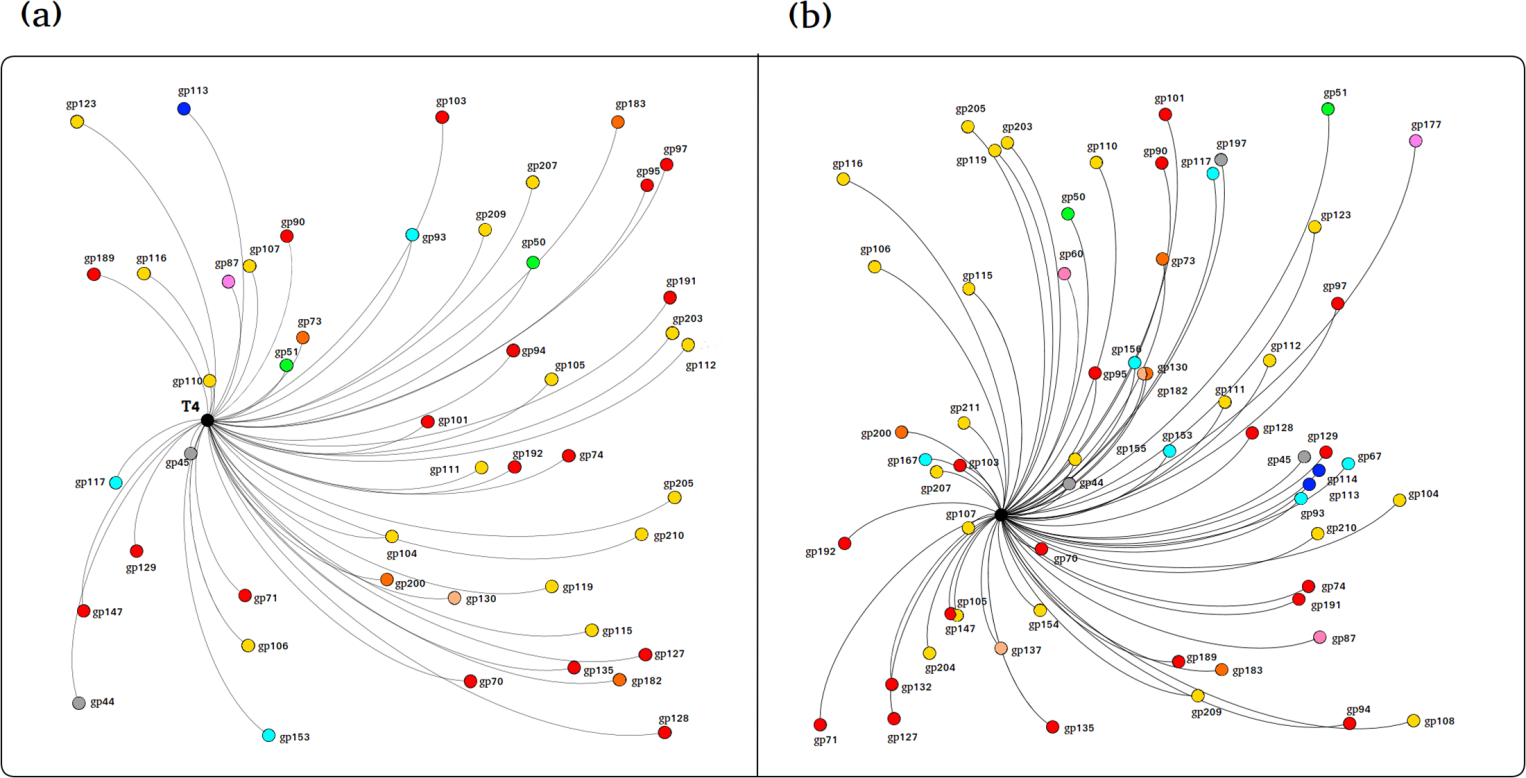
Network representations of the BLASTx based core gene analysis of pf16 and the putative *Tevenvirinae*. (a) pf16 BLASTx network of genes related to *Enterobacteria* phage T4 alone. (b) pf16 BLASTx network of genes related to the putative *Tevenvirinae* following “gene filtration” via BLASTx analysis of all genes against T4 followed by compilation into a new database. T4 is represented as the black dot in the centre of each network. Genes are coloured according to predicted function as per legends provided for Figs 2 and 3. Gene product (gp) labels are provided at each locus. Distance from T4 central dot correlates with relatedness of gp relative to other genes.

To investigate the core genome possibility, all 156 putative *Tevenvirinae* genomes were analysed to identify whether any regions are common to all phages including pf16. This is much larger than previous studies [89, 90]. Gene matrix plots highlighting the presence and absence of core and accessory genes within a majority of genomes do show that there is some level of conservation across the phages (Fig 8a). Plotting the number of unique genes against number of genomes reveals a steady rise with some levelling off. The overall pan-genome of the phages shows a fairly consistent rise, highlighting the extensive diversity of the many accessory genes; however, and perhaps unsurprisingly, the number of completely new genes declines with genome number (Fig 8b, 8c and 8d). This shows that whilst the majority of genomes contain some genes not found in other phages and that the putative *Tevenvirinae* have an impressive array of AMGs at their disposal, the overall number of new genes of equivalent function gradually decreases, implying a level of commonality amongst a majority of the putative *Tevenvirinae*. Ultimately to determine the relatedness of pf16 to the putative *Tevenvirinae*, a BLAST based core gene analysis was carried out. From the T4 analysis alone, 47 matches were obtained, given as a network representation in Fig 9. 17 of these are genes involved in DNA replication/recombination/repair, including the DNA polymerase, helicase/primase, UvsW, UvsX, UvsY, and topoisomerase subunits. 16 hits are structural constituents, important matches including the major capsid protein and several baseplate and tail constituents. Other notable hits include the terminase large subunit, several proteins involved in nucleotide metabolism, homing endonucleases, and even two hypothetical proteins (gp44 and gp45). When we subsequently look at the filtered BLAST against all putative *Tevenvirinae*, 61 hits were obtained (Fig 9). Of the 14 additional hits, 5 of these are proteins without equivalents in T4 even in the absence of homology, these are a swarming motility-like protein (gp60), PIF1-like helicase (gp132), FmdB-like regulatory protein (gp137), putative glutaredoxin (gp177), and a hypothetical protein (gp197). What this leaves are 6 structural proteins mainly for the tail and baseplate, a homing endonuclease, RNA ligase, and the terminase small subunit. Together, this means that 25.6% of the genes encoded by pf16 have equivalents in T4 detectable using homology based approaches. This analysis was unable to pick up some genes such as the DsbA equivalent in pf16 (gp68) so in fact the number of shared genes is in reality slightly higher.

T4, *Vibrio* phage KVP40, and the *Acinetobacter* phage ZZ1 were shown to have a tendency to evolve via gene duplication events. [65, 91, 92]. It seems that gene duplication, insertions, and subsequent divergence is an important, potentially characteristic mechanism of T4-like evolution. This was investigated in pf16 by BLASTp analysis against itself followed by confirmation of putative duplications (those with >20% identity) by analysis at the nucleotide level. Only those showing nucleotide homology were taken to be putative paralogs. From this analysis, 7 putative gene duplications were found in pf16. These are gp10/11, gp27/49, gp82/83, gp94/118, gp152/153, gp169/170, and gp202/203. Whilst most candidates encode hypothetical proteins, gp82 is a putative GTP Cyclohydrolase I, whilst its paralog is a hypothetical protein within the queuosine module. Interestingly, gp94 encodes a deoxyribonucleotidase component, whilst its paralog gp118 encodes a neck protein, showing a stark example of gene duplication and divergence to very different functions. The same analysis on phiPMW yielded no putative duplications.

To deduce the phylogenetic relationships within the putative *Tevenvirinae*, and pf16's placement within the group, a composite Bayesian phylogenetic tree was constructed from concatenated alignments of the MCP, replicative helicase, and TerL proteins. S3 Fig shows the resulting relationships. It can be seen that the *Tevenvirinae* and candidate members are composed of four distinct clusters. Despite some homology in specific genes to cyanophages, pf16 occupies a cluster at the interface of the Schizo T-Even and Exo T-Even viruses which we define here as the “pf16-related” phages, showing similarity to *Ralstonia* phage RSP15 (LC121084.1). The *Rhodothermus marinus* phage RM378 occupies an isolated branch on the tree [93].

Taking together the morphological similarity, core gene analysis, and phylogenetic relationships, we propose that pf16 at least assumes status as a peripheral member of the *Tevenvirinae*. This is due, in part, to the fact that this group of phages at the time of writing prove difficult to classify. We believe that all phages analysed here merit inclusion in some regards to the group (with the possible exception of RM378) but that solid classification probably will not come until a minimum genome for T4-related phages is truly defined and a cut-off established.

#### phiPMW

With respect to phiPMW, of the 90 ORFs which show some homology (E-value <0.01), 14 of these are related to various “Felixounavirinae”, including the MCP and TerL [13]. In addition, the ribonucleotide reductase alpha and beta subunits, nicotinamide phosphoribosyltransferase, and an RNA ligase exhibiting adenylyltransferase activity, proteins typically found in the “Felixounavirinae”, all have an origin within this subfamily in phiPMW. This homology is observable in Fig 3 when phiPMW is compared to *P*. *aeruginosa* phages PAK_P1 and JG004. phiPMW's 6 tRNAs, genome size of 103,218 bp, and GC% of 45.15%, is most closely matched by members of the KIL-like viruses and indeed a number of ORFs within phiPMW are related to phiPSA374 and VCM (Rombouts et al. 2016). As stated above, no gene duplication events were detectable within phiPMW however, a number of ORFs show homology to other *Pseudomonas* phages. In particular, there are 10 ORFs related to the *P*. *tolaasii* siphovirus phiPto-bp6g; a completely unrelated phage [94]. This, amongst other genes, suggests that phiPMW may have evolved primarily through the acquisition of genes from external sources followed by their extensive divergence, in contrast to the gene duplication seen in pf16.

Composite Bayesian phylogenetic analysis was carried out for phiPMW against the rest of the “Felixounavirinae” and related rv5-like viruses [95–97]. From the resulting tree (S4 Fig), we can clearly see the *Pakpunaviruses*, *Kpp10viruses*, KIL-related, FelixO1-related, and *Vequintavirinae* virus groups occupying five very distinct clusters as described previously [13]. As expected, the rv5-like viruses occupy the most distant group outside the proposed “Felixounavirinae” classification. Interestingly, phiPMW occupies a lone branch between the “Felixounavirinae”-infecting *Pseudomonas* species and those related to *Salmonella* phage FelixO1. Taking into account only typically conserved proteins, one could conclude the phiPMW resides as a distant member of the “Felixounavirinae”, however due to the extensive novelty observed in the phiPMW genome we argue that it is far too distinct to merit inclusion into this group and that it is in fact a novel phage albeit with an evolutionary history involving the “Felixounavirinae”.

## Conclusions

In this work, two novel phages infecting *P*. *Putida* were characterised. Pf16 represents a taxonomically distinct and proposed peripheral T4-related phage. Phylogenetic analysis demonstrated that pf16 occupies a clade defined here as the pf16-related phages, yet evidence suggests that pf16 has its origins rooted within the cyanophages. Pf16 encodes an analogue of the stringent starvation protein B which may play a role in host biofilm regulation. It was discovered that phiPMW represents a novel type of phage with an evolutionary history involving the “Felixounavirinae”. The phiPMW genome proved to be highly enigmatic and evolved via the acquisition of genes of external origin followed by extensive divergence.

One thing for sure has been gained from the analysis of both pf16 and phiPMW, and that is that they have highlighted two persistently major problems within the field of viral taxonomy. The first is evident with pf16 and that is in defining what the threshold is for inclusion of a phage within a particular group. When incorporating metagenomic datasets, the phylogenetic clustering of all putative *Tevenvirinae* is achieved however, the true utility of conventional phylogenetic markers such as the major capsid protein and the terminase large subunit is clearly questionable when we look at phiPMW. There is obviously no panacea when it comes to viral taxonomy and it is likely that the only way forward will be to utilise complete genomic and physical information of phages as well as establish clear, yet flexible membership criteria for inclusion into groups.

### Accession numbers

Bacteriophages pf16 and phiPMW are deposited on NCBI under the accession numbers KU873925 and KU862660.

## Supporting information

S1 Fig Web logo representation of pf16 and phiPMW promoter elements. (a) pf16 phage specific promoter. (b) phiPMW putative operon promoter. Height/size of letters corresponds to conservation of that particular base in the sequence.
(PNG)

S2 Fig Graph showing percentage of codons in ORFs from *Pseudomonas* phages pf16 and phiPMW recognised by phage encoded tRNAs. pf16 is represented by (**X**) and phiPMW as (**.**). A 20% threshold is represented by the horizontal line.
(PNG)

S3 Fig Bayesian phylogenetic tree of the *Tevenvirinae*. Created using concatenated alignments of the major capsid, terminase large subunit, and replicative helicase proteins and generations run until an average standard deviation of split frequencies of under 0.01 was achieved. The previously characterised Exo-T-Even, T & Pseudo-T-Evens, and Schizo-T-Even groups, are coloured and labelled accordingly. Pf16 clusters into a newly defined clade known as the pf16-like T-Evens (in dark blue) whilst *Rhodothermus* phage RM378 occupies an isolated branch in the tree.
(PNG)

S4 Fig Bayesian phylogenetic tree of the “Felixounavirinae” and rv5 -related viruses of the *Vequintavirinae*. Created using concatenated alignments of the major capsid, terminase large subunit, and replicative helicase proteins and generations run until an average standard deviation of split frequencies of under 0.01 was achieved. The *Pakpunaviruses*, *Kpp10viruses*, KIL-related, FelixO1-related, and rv5-related *Vequintavirinae* viruses are all coloured and labelled accordingly with phiPMW in dark blue.
(PNG)

S1 Table Table showing *Pseudomonas* phage pf16 ORFs and corresponding characteristics. (XLSX)

S2 Table Table showing *Pseudomonas* phage phiPMW ORFs and corresponding characteristics. (XLSX)

S3 Table Codon usage patterns across *Pseudomonas putida* KT2440 and bacteriophages pf16 and phiPMW. Percentages corresponding to codons recognised by phage encoded tRNAs are highlighted in bold and underlined.
(PDF)
