# Supplementary figures and images for: Pf16 and phiPMW: Expanding the realm of Pseudomonas putida bacteriophages
Source: PLoS One. 2017 Sep 6;12(9):e0184307. doi: 10.1371/journal.pone.0184307 (PMC5587285; doi:10.1371/journal.pone.0184307)

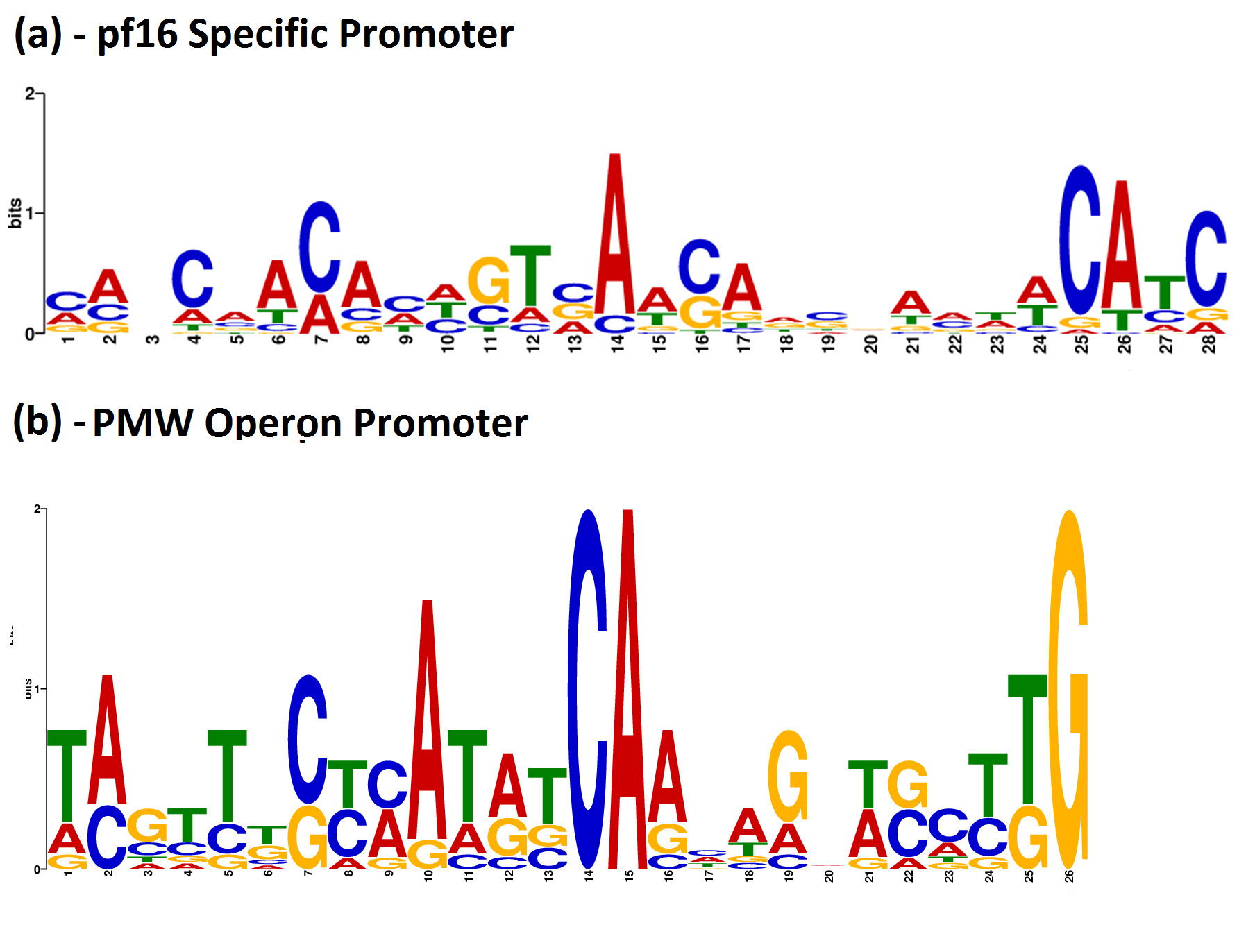

Supplement: S1 Fig — (a) pf16 phage specific promoter. (b) phiPMW putative operon promoter. Height/size of letters corresponds to conservation of that particular base in the sequence. (PNG) [file pone.0184307.s001.png]

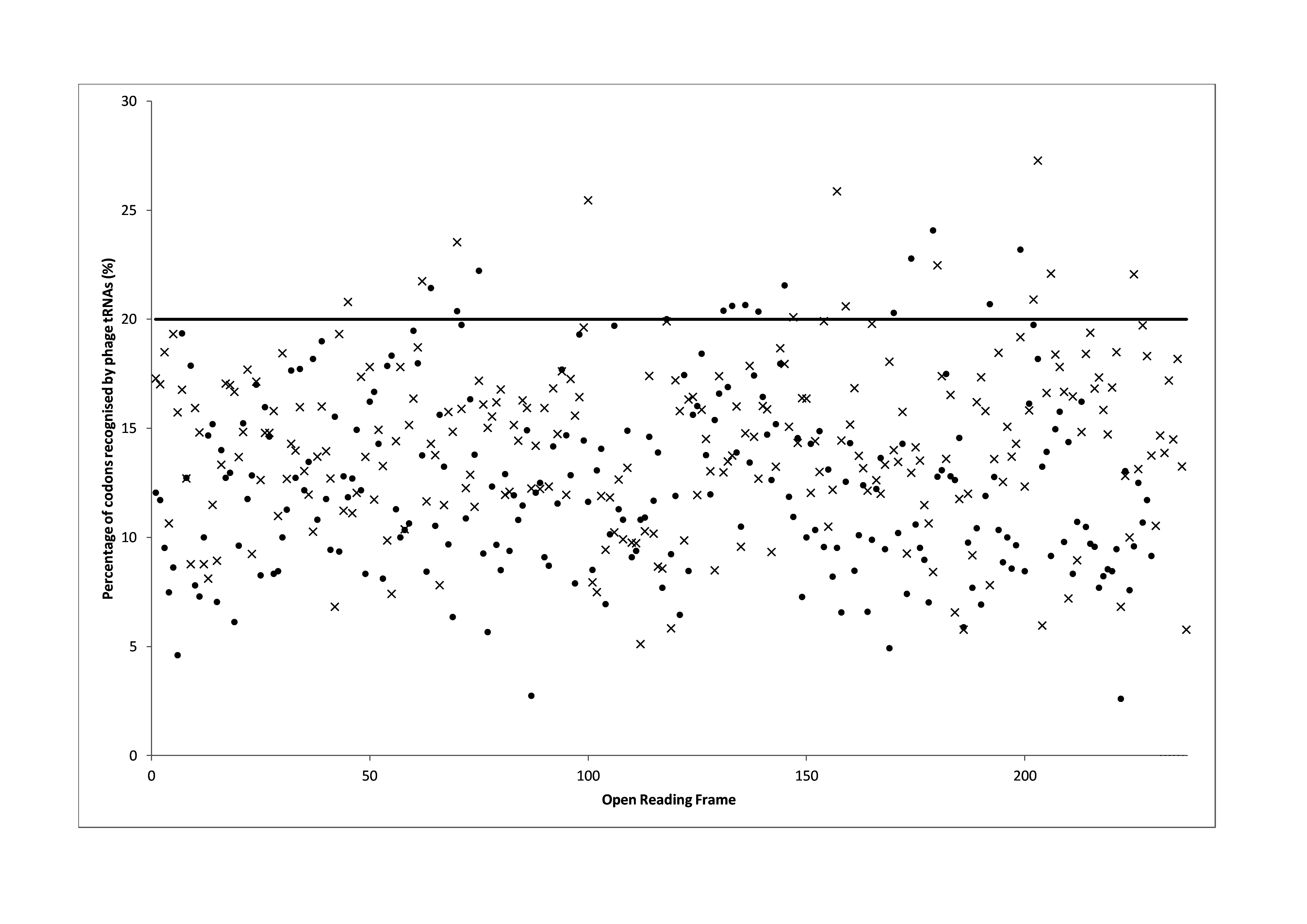

Supplement: S2 Fig — pf16 is represented by (X) and phiPMW as (.). A 20% threshold is represented by the horizontal line. (PNG) [file pone.0184307.s002.png]

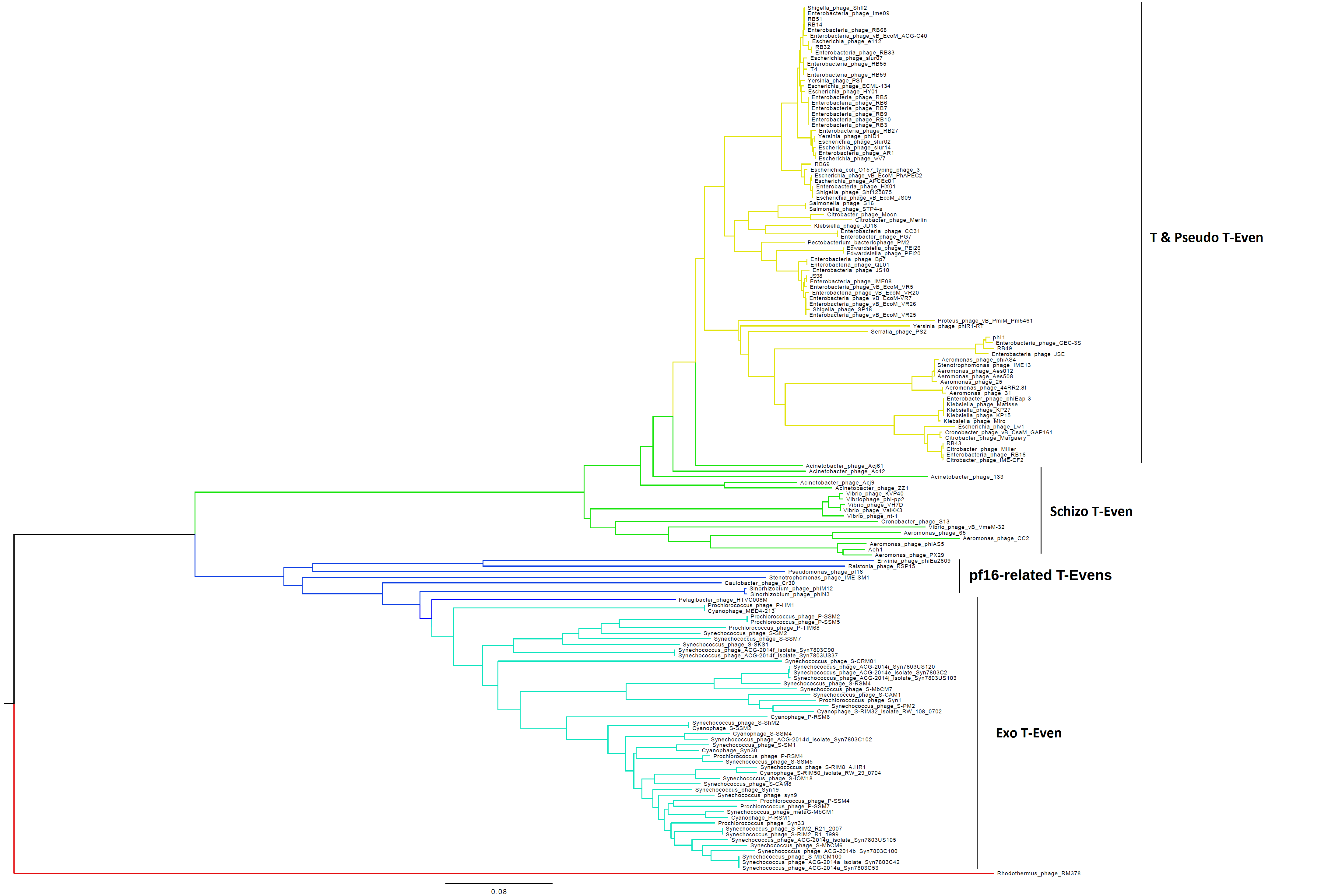

Supplement: S3 Fig — Created using concatenated alignments of the major capsid, terminase large subunit, and replicative helicase proteins and generations run until an average standard deviation of split frequencies of under 0.01 was achieved. The previously characterised Exo-T-Even, T & Pseudo-T-Evens, and Schizo-T-Even groups, are coloured and labelled accordingly. Pf16 clusters into a newly defined clade known as the pf16-like T-Evens (in dark blue) whilst Rhodothermus phage RM378 occupies an isolated branch in the tree. (PNG) [file pone.0184307.s003.png]

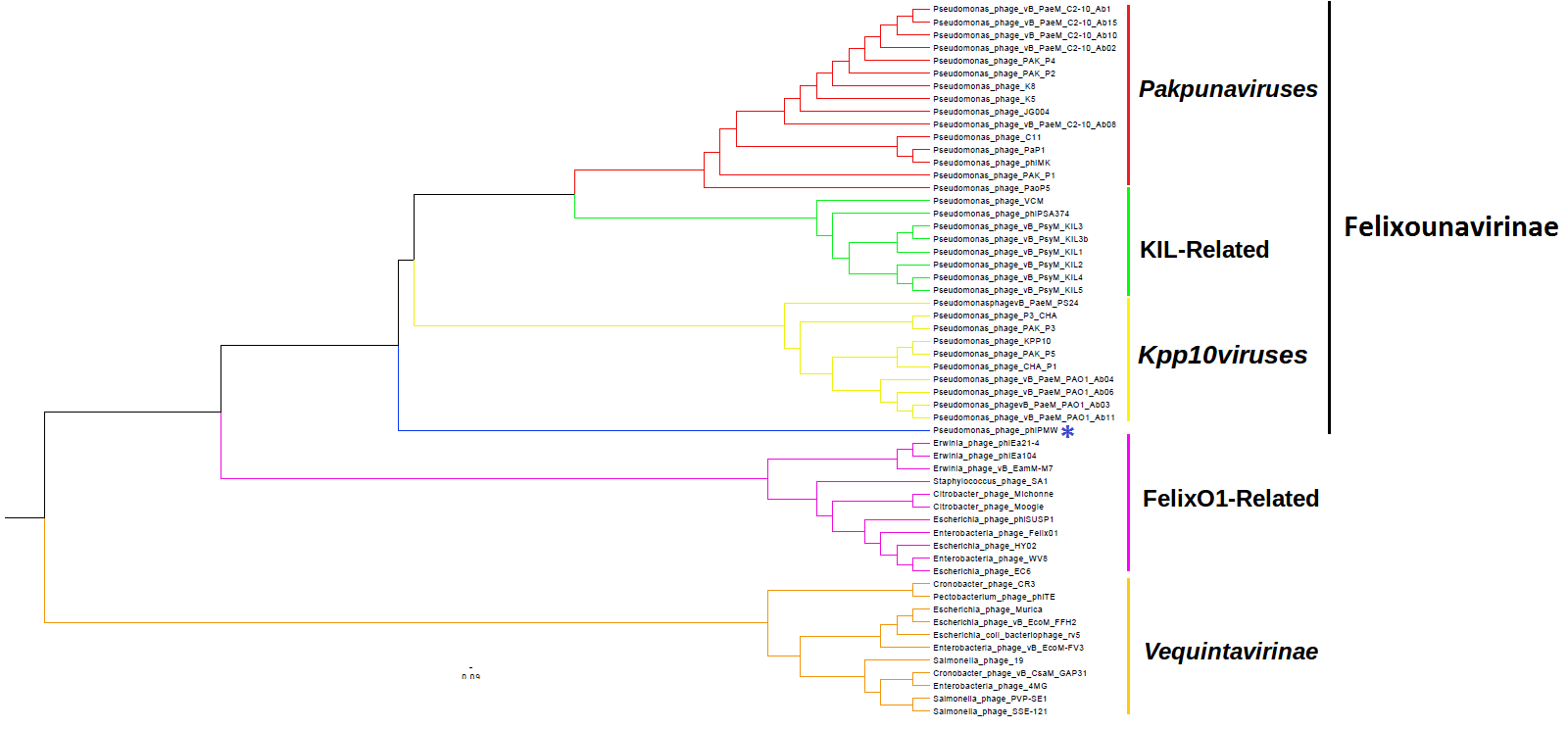

Supplement: S4 Fig — Created using concatenated alignments of the major capsid, terminase large subunit, and replicative helicase proteins and generations run until an average standard deviation of split frequencies of under 0.01 was achieved. The Pakpunaviruses, Kpp10viruses, KIL-related, FelixO1-related, and rv5-related Vequintavirinae viruses are all coloured and labelled accordingly with phiPMW in dark blue. (PNG) [file pone.0184307.s004.png]
