# Supplementary material for: Pf16 and phiPMW: Expanding the realm of Pseudomonas putida bacteriophages
Source: PLoS One. 2017 Sep 6;12(9):e0184307. doi: 10.1371/journal.pone.0184307 (PMC5587285; doi:10.1371/journal.pone.0184307)
Supplement: S3 Table — Percentages corresponding to codons recognised by phage encoded tRNAs are highlighted in bold and underlined. (PDF) [file pone.0184307.s007.pdf]

Table S5. Codon usage patterns of *Pseudomonas putida* KT2440 and bacteriophages pf16 and phiPMW: Percentages corresponding to codons recognised by phage encoded tRNAs are highlighted in bold and underlined.

Codon	Percentage of *Pseudomonas putida* Codons	Percentage of pf16 Codons	Percentage of phiPMW Codons	pf16 Codons Descending Order (%)	phiPMW Codons Descending Order (%)
CTG	7.16	1.87	2.22	2.77	3.00
GCC	5.67	1.67	0.84	2.70	2.67
GGC	4.89	1.62	0.73	2.56	2.45
GAC	3.65	1.77	1.45	2.30	2.38
GTG	3.62	1.75	1.61	2.28	2.34
CGC	3.60	1.54	0.71	2.25	**<u>2.29</u>**
CAG	3.51	2.56	1.59	2.13	2.22
ATC	3.44	1.70	**<u>1.71</u>**	2.10	2.15
ACC	3.10	1.74	1.61	2.08	2.12
GAG	2.96	1.38	1.49	2.00	**<u>2.12</u>**
GCG	2.83	1.83	0.67	**<u>1.98</u>**	2.05
TTC	2.82	2.25	**<u>2.12</u>**	1.90	**<u>2.05</u>**
GAA	2.71	1.77	2.38	1.87	2.04
AAG	2.45	1.60	2.34	1.87	2.02
CCG	2.43	1.11	0.76	**<u>1.86</u>**	1.98
AAC	2.33	**<u>1.86</u>**	**<u>2.05</u>**	1.86	1.94
AGC	2.31	2.13	1.31	**<u>1.86</u>**	**<u>1.85</u>**
ATG	2.31	**<u>1.77</u>**	2.12	1.85	1.84

GTC	2.14	1.87	1.16	1.84	1.83
TAC	1.83	**<u>1.30</u>**	1.48	1.83	1.80
GAT	1.66	1.90	1.84	1.81	1.78
TTG	1.64	2.70	2.45	1.81	1.77
GGT	1.64	1.71	2.04	1.77	1.76
CAC	1.55	1.86	1.60	1.77	1.73
CTC	1.49	1.44	1.29	**<u>1.77</u>**	1.72
TGG	1.44	**<u>1.86</u>**	**<u>1.85</u>**	1.75	**<u>1.71</u>**
TCG	1.39	1.81	1.18	1.74	**<u>1.71</u>**
GCA	1.37	1.85	1.80	1.72	1.70
CGT	1.27	1.45	1.29	1.71	1.65
GCT	1.21	1.84	1.76	1.70	1.61
GGG	1.17	0.98	0.87	1.69	1.61
CCC	1.14	0.90	0.76	1.67	1.60
CAA	1.09	1.66	3.00	1.66	1.59
CGG	1.03	1.24	0.71	1.66	1.58
AAA	0.99	**<u>0.90</u>**	2.15	1.63	1.50
ATT	0.97	0.62	1.83	1.62	1.49
ACG	0.89	1.69	1.19	1.60	1.48
TCC	0.87	1.07	1.14	1.54	1.46
TGC	0.86	2.10	1.50	**<u>1.54</u>**	1.45
CAT	0.81	1.72	1.73	1.50	1.33

GTA	0.75	1.50	1.94	1.46	1.31
GTT	0.75	2.08	1.98	1.45	1.31
TTT	0.73	1.19	1.46	1.44	1.29
CTT	0.73	2.30	2.02	1.40	1.29
TAT	0.72	0.57	1.23	1.38	1.25
CCA	0.71	**1.98**	**1.71**	**1.30**	1.23
AAT	0.67	0.63	1.78	1.29	1.19
CCT	0.60	1.46	1.02	1.24	1.18
AGT	0.52	1.66	1.77	1.19	1.18
ACT	0.47	1.40	1.72	1.13	1.16
ACA	0.36	**1.54**	**2.29**	1.12	1.14
CGA	0.34	2.00	1.11	1.11	1.14
GGA	0.33	1.13	1.31	1.07	1.11
TCA	0.30	**2.77**	2.05	0.98	1.03
CTA	0.27	0.56	1.03	0.90	1.02
TCT	0.27	1.29	1.58	0.90	0.87
AGG	0.23	1.63	1.25	**0.90**	0.84
ATA	0.21	0.78	1.14	0.78	0.77
TGT	0.21	1.81	1.70	0.63	0.76
TGA	0.20	2.28	2.67	0.62	0.76
TTA	0.15	0.56	1.18	0.57	0.73
AGA	0.12	1.12	1.65	0.56	0.71

TAA	0.07	0.52	1.33	0.56	0.71
TAG	0.04	0.90	0.77	0.52	0.67
